# Ovarian Cancer: A Landscape of Mitochondria with Emphasis on Mitochondrial Dynamics

**DOI:** 10.3390/ijms24021224

**Published:** 2023-01-08

**Authors:** Domenico De Rasmo, Antonella Cormio, Gennaro Cormio, Anna Signorile

**Affiliations:** 1 Institute of Biomembranes, Bioenergetics and Molecular Biotechnology (IBIOM), National Research Council (CNR), 70124 Bari, Italy; 2 Department of Precision and Regenerative Medicine and Ionian Area, University of Bari Aldo Moro, 70124 Bari, Italy; 3 IRCCS Istituto Tumori “Giovanni Paolo II”, 70124 Bari, Italy; 4 Department of Interdisciplinary Medicine, University of Bari Aldo Moro, 70124 Bari, Italy; 5 Department of Translational Biomedicine and Neuroscience, University of Bari Aldo Moro, 70124 Bari, Italy

**Keywords:** ovarian cancer, mitochondria, mitochondrial dynamics, OPA1, DRP1, MFN2, prohibitin, cAMP/PKA

## Abstract

Ovarian cancer (OC) represents the main cause of death from gynecological malignancies in western countries. Altered cellular and mitochondrial metabolism are considered hallmarks in cancer disease. Several mitochondrial aspects have been found altered in OC, such as the oxidative phosphorylation system, oxidative stress and mitochondrial dynamics. Mitochondrial dynamics includes cristae remodeling, fusion, and fission processes forming a dynamic mitochondrial network. Alteration of mitochondrial dynamics is associated with metabolic change in tumour development and, in particular, the mitochondrial shaping proteins appear also to be responsible for the chemosensitivity and/or chemoresistance in OC. In this review a focus on the mitochondrial dynamics in OC cells is presented.

## 1. Introduction

Ovarian cancer (OC) represents the leading cause of death from gynaecological malignancies in western countries. The absence of specific symptoms at the early stage of the disease, and the available diagnostic test for OC detection, lead to a delay of the diagnosis [1] maintaining the mortality rate [2]. The majority of OC are epithelial-derived tumours and exhibit several histopathological phenotypes defined as serous, mucinous, endometrioid, clear cell, squamous, mixed and undifferentiated types [3]. Moreover, serous ovarian carcinoma can be subdivided into high-grade and low-grade cancers. The latter usually evolve from adenofibromas or borderline tumors, have frequent mutations of the KRAS, BRAF, or ERBB2 genes, lack TP53 mutations, have an indolent behaviour and respond less to platin and taxol chemotherapy [4]. In the recent years, in addition to these classifications, data based on genomic signatures such as mutations in the BRCA1 or BRCA2 genes or methylation of the BRCA1 or RAD51C promoters, highlight the existence of others molecular subgroups [5,6] that differently respond to therapy [7,8]. Moreover, transcriptomic profiling has allowed the identification of additional molecular subtypes [9,10,11]. Despite all these classifications, the standard treatment of OC consists of tumour debulking surgery followed by platinum and taxane-based therapy. Patients typically tolerate the treatment well and go into remission, but due to the advanced-stage of diagnosis, disease recurrence and chemotherapy-resistance are complications that often arise during the disease.

In general, altered cellular metabolism and mitochondrial deregulation are considered hallmarks for the onset and evolution of cancer disease [12,13,14]. The mitochondria are the core of cellular energy metabolism, oxidative stress, and cell signalling. All these aspects have been found altered in cancer, and are closely associated with cancer development [15,16,17]. Several studies have found that mitochondrial dysfunctions are extensively and directly implicated in OC [18,19,20]. Scanning electron microscope analysis of ovarian cancer tissues demonstrates an increased mitochondrial number, and mitochondria maximum length, as well as a reduction of cristae width and junction diameter [21,22] that can influence mitochondrial bioenergetics [23]. Moreover, different mitochondrial genetic alterations, such as changes in mitochondrial DNA (mtDNA) content and mtDNA mutations, have been reported in OC and are often associated with oxidative phosphorylation (OXPHOS) system deregulation [24,25]. The processes of cristae remodelling, fusion, and fission of mitochondria, is called mitochondrial dynamics [26]. Mitochondria continuously divide and fuse forming a dynamic mitochondrial network providing an adaptation to metabolic changes, preserving cell integrity, and protecting against autophagy [27]. Mitochondrial dynamics and, in particular, mitochondrial shaping proteins appear also to be responsible for the chemosensitivity and/or chemoresistance in different gynaecological cancers including OC [28]. In this review a focus on the mitochondrial dynamics deregulation in OC, also related to chemoresistance and chemosensitivity, is presented.

## 2. Mitochondrial Overview in Cancer

**Figure 1:**
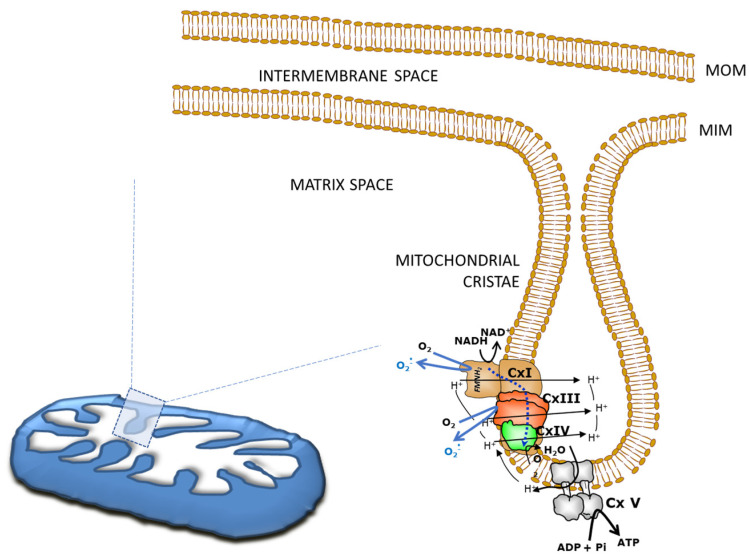
Schematic representation of mitochondrial structures. Mitochondria have a mitochondrial outer membrane (MOM), an intermembrane space, and a mitochondrial inner membrane (MIM). The inner membrane borders the mitochondrial matrix and forms mitochondrial cristae. The enzymes of mitochondrial respiration chain, complexes I, III and IV (CxI, CxIII, CxIV) and ATP synthase (Cx V) are localized in the MIM. The electron transfer through the mitochondrial respiratory complexes is coupled to the proton transfer (H^+^) from the matrix to the intermembrane space generating a mitochondrial membrane potential used for releasing ATP from CxV. Mitochondria complexes I and III generate oxygen reactive species (O_2_^.^) [30].

A mitochondrion has an outer membrane, an intermembrane space, and an inner membrane. The inner membrane delimits the mitochondrial matrix and forms mitochondrial cristae containing the enzymes for mitochondrial respiration and ATP production (Figure 1). The mitochondrion is considered the “powerhouse” of cells producing more than 90% of ATP. Electron transfer through the mitochondrial respiratory complexes is coupled to the proton transfer from the matrix to the intermembrane space generating an electrochemical gradient, which provides energy for releasing ATP from F1Fo-ATP synthase (Figure 1) [29,30].

Moreover, in the mitochondria other metabolic pathways such as the urea cycle and fatty acid β-oxidation take place. In addition, mitochondria physiologically generate oxygen reactive species (ROS), and they possess an efficient antioxidant system to control the redox homeostasis [31]. Mitochondrial ROS participate in cellular signalling but an increase of ROS production (oxidative stress) is involved in the pathogenesis of several diseases [32,33,34,35], including cancer [36,37].

**Figure 2:**
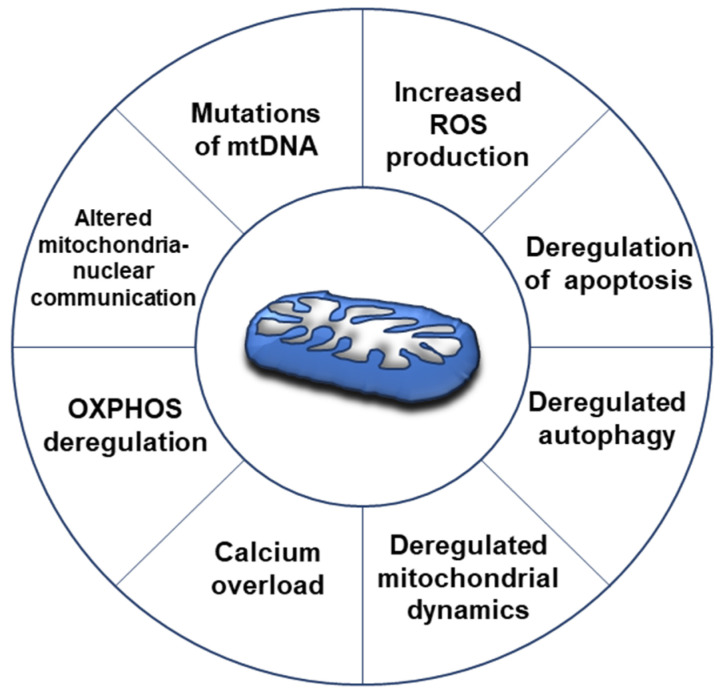
Mitochondrial alterations in cancer. Several aspects have been found altered in cancer cells, such as mtDNA mutation, mitochondria-nuclear communication, oxidative stress, cell apoptosis, autophagy, dynamics and calcium overload [38,39,40]. To sustain cell proliferation, the cancer cells acquire the ability to change their metabolism equilibrating both glycolysis and OXPHOS for ATP production [43,44].

Many mitochondrial aspects have been found altered in cancer, such as mtDNA mutation, mitochondria-nuclear communication, oxidative stress, cell apoptosis, autophagy, dynamics and calcium overload (Figure 2) [38,39,40]. In particular, in different cancer diseases [41], emerging evidence highlights that deregulation of mitochondrial dynamics is involved in several aspects of cancer development, such as cancer metastasis, drug resistance and cancer stem cell survival. Thus, mitochondrial shaping proteins and their regulators have been proposed as potential targets for therapeutic approaches [41,42].

All mitochondrial aspects can be modulated by different cellular pathways, resulting in increase of complexity of picture. To sustain cell proliferation and their existence in the body, the cancer cells acquire the ability to change their metabolism in a hybrid metabolic phenotype equilibrating both glycolysis and OXPHOS for ATP production [43]. The metabolic reprogramming, fluctuations in bioenergetic fuels, and the modulation of oxidative stress are key hallmarks of cancer development. In cancer cells, elevated glucose uptake and high glycolytic rate, as a source of ATP, constitute a growth advantage and represent the universally known as the Warburg effect [45]. However, glucose utilization and carbon sources in cancer are much more heterogeneous than initially thought. Indeed, new studies have revealed a dual capacity of cancer cells to use both glycolytic and oxidative metabolism to sustain ATP production [46]. In addition, tumours sharing the same clinical diagnosis can show mitochondrial metabolic heterogeneity using glutamine or fatty acids as alternative oxidizable substrates [47]. Metabolic reprogramming supports cancer cell proliferation, survival, migration, and invasion. Moreover, ROS produced by mitochondrial metabolism and nutrient availability are important for interaction with cancer microenvironment components [44].

Mitochondria are also key regulators of apoptosis. Mitochondria-mediated caspase activation pathway is a major apoptotic pathway characterized by mitochondrial outer membrane permeabilization and subsequent release of cytochrome c into the cytoplasm to activate caspases [48]. Mitochondrial membrane integrity is tightly regulated by the balance and interaction of anti-apoptotic BCL-2 and proapoptotic BAX and BAK proteins. Studeis have shown interactions between BAK and BCL-2 with the proteins involved in mitochondrial dynamics, supporting the finding of intense crosstalk between mitochondrial dynamics and apoptosis machineries [28]. Deregulated apoptosis can be responsible for enhanced apoptosis resistance of cancer cells, supporting a high proliferation rate and drug resistance [49,50,51].

## 3. Mitochondrial Alterations in Ovarian Cancer

Several genomic and proteomic studies have shown mitochondrial disfunctions in OC [52,53,54]. Regarding DNA mutations, in OC up to 60% are found in mtDNA, particularly in the D-loop region, 12 S, 16 S rRNA and cytochrome b genes [53,55]. Increased mtDNA in ovarian cancer with respect to control tissues has been reported and, further, the mtDNA content in low-grade tumours is over two-fold higher than that in high-grade carcinomas [56].

The mitochondrial genetic alteration in OC has often been associated with OXPHOS deregulation [24,25]. The increase of mtDNA copy number in ovarian cancer cells and tissues could suggest the need of a sustained mitochondrial function for tumour growth [21,56]. In fact, ovarian cancer cells can exhibit sustained oxidative mitochondrial activities in terms of membrane potential, ATP synthesis and oxygen consumption [57,58]. In addition, Signorile et al. reported an increase of respiratory chain complex activities and citrate synthase in human ovarian cancer tissues [21] associated with an increase of mitochondrial number and mtDNA. This appears to be due to an increase in cAMP level that, in turn, can induce the PGC-1α expression and thus mitochondrial biogenesis [21]. However, in the same samples a decrease of complex I activity has been reported despite the increase of mitochondrial biogenesis [21]. Additionally, in an ovarian cancer cell model, the induction of respiratory complex I impairment by genetic ablation or inhibitors elicits an increase in PGC-1α expression associated with increase of ROS production [59]. In this case the authors attributed the increase expression of PGC-1α to the enhanced cellular ROS level [59].

Metabolic heterogeneity represents a very important aspect of ovarian cancer cells. In fact, bioenergetic analyses defined two molecular subgroups for ovarian cancer cells, one with low and another one with high OXPHOS activity. High-OXPHOS tumours are characterized by upregulation of genes encoding for respiratory chain proteins with respect to low OXPHOS cells, and are associated with increased mitochondrial respiration and enhanced antioxidant defences. These aspects were found to be related to stress-mediated promyelocytic the leukemia protein-peroxisome proliferator-activated receptor gamma coactivator-1a (PML-PGC-1a) axis. Importantly, high-OXPHOS cells exhibit increased chemosensitivity to ROS-producing agent therapy [60]. Accordingly, in drug-resistant ovarian cancer cells, high levels of complex III of the mitochondrial respiratory chain are associated with high sensitivity to complex III inhibition [61].

In ovarian cancer cells, the alteration of mitochondrial respiratory chain complexes has been associated with the deregulation of mitochondrial biogenesis [21]. Mitochondrial biogenesis can be defined as the growth and division of pre-existing mitochondria, and this process involves integration of several signals and proteins. PGC-1α is a co-transcriptional regulation factor that induces mitochondrial biogenesis by activating different transcription factors, including NRF-1 and NRF-2, which promote the expression of TFAM which drives transcription and replication of mtDNA [62]. An increase of PGC-1α and TFAM expression proteins has been found in ovarian cancer cells and tissues [21,59], which is consistent with the increase of mtDNA content and mitochondrial number [21,56]. The same tissues showed an increased level of prohibitin proteins [21]. Prohibitin 1 (PHB1) and prohibitin 2 (PHB2) are proteins ubiquitously expressed that have a critical role within mitochondria. In mitochondria, PHB1 and PHB2 assemble at the inner membrane to form a supra-macromolecular structure that works as a scaffold for proteins and lipids regulating mitochondrial metabolism, including bioenergetics, biogenesis, and dynamics in order to determine the cell fate [63]. Prohibitins have been found to promote, by PGC-1α and TFAM, the expression of proteins encoded by both nuclear and mitochondrial DNA [64,65]. Furthermore, PGC-1α expression can also be regulated by activation of the cAMP/PKA pathway [66], which is altered in ovarian cancer tissues, presenting increased level of cAMP and increased PKA activity [21].

## 4. Mitochondrial Dynamics in Ovarian Cancer

### 4.1. Proteins Involved in Mitochondrial Dynamic Machinery

Mitochondria are highly dynamic organelles, continuously join by the process of fusion, and divide by the process of fission. Mitochondrial dynamics are directly linked to modulation of mitochondrial physiology in order to adapt cell to metabolic changes and preserve cell integrity [27]. These processes involve different proteins and regulatory signalling pathways [67,68,69,70]. The fusion process is mediated mainly by the dynamin-related proteins mitofusin-1 (MFN1), mitofusin-2 (MFN2) and optic atrophy 1 (OPA1).

MFN1 and MFN2 have similar structural organization [71]; however their specific role in the fusion process may be different. In fact, MFN2 has been shown to participate in interactions between mitochondria and between mitochondria and other organelles, in particular with the endoplasmic reticulum. MFN1 interacts physically with MFN2 [72] and with OPA1 [73]. OPA1 is a protein localized in the inner mitochondrial membrane and intermembrane space where it is responsible for mitochondrial fusion, cristae remodelling and apoptosis [74,75,76]. The OPA1 structure shows a dynamin portion formed by GTPase activity, a middle lipid-binding and GTPase effector domains. The expression of OPA1 is regulated by alternative splicing mechanism resulting in eight different protein isoforms [77]. In addition, OPA1 function is regulated by constitutive proteolytic processing that produces a long and membrane-bound form L-OPA1, and a short and soluble form S-OPA1 [78].

The balance between these two isoforms is involved in the mitochondrial network and apoptosis [79]. L-OPA1 and S-OPA1 both can regulate mitochondrial morphology [75]. In particular, L-OPA1 is mainly responsible for MIM fusion, and S-OPA1 further facilitates mitochondrial fission. Both forms are involved in cristae structure organization [80]. The proteases responsible for its processing are YME1L, OMA1 peptidases and PARL [80,81]. The constitutively activity of YME1L can also be modulated by different post-translational modification and/or signalling pathways, such as the SIRT3-mediated deacetylation, that suppresses its activity on OPA1 cleavage and thus facilitate mitochondrial fusion [82]. OMA1 is a stress-activated peptidases that promotes rapid proteolytic conversion of L-OPA1 to the S-OPA1 forms, resulting in fragmentation of the mitochondrial network [83,84]. Interestingly, OMA1 is also important for stability of cristae junction organizing system (MICOS). In fact, OMA1 can associate with MICOS through the MIC60 subunit. The interaction between OMA1 and MICOS is required for optimal bioenergetic output and apoptosis [85]. The rhomboid protease PARL participates in the production of S-OPA1 and itself is regulated by proteolysis to generate a cleaved form, which in turn modulates the shape of mitochondria [86]. PARL participate in a large protease complex with SLP2 and YME1L that represents a large proteolytic hub for coordination of proteolytic functions in the inner mitochondrial membrane [87].

The proteolytic processing of OPA1 is also regulated by post translational modification, such as by acetylation [69].

Mitochondrial fission is a process that cells adopt to distribute and reorganize their mitochondrial network [88]. The most important proteins involved in mitochondrial fission are dynamin-1-like protein (DRP1) and mitochondrial fission 1 protein (FIS1). FIS1 is mainly located in the mitochondrial outer membrane. DRP1 moves between the cytosol and the outer mitochondrial membrane where it participates in the constriction of the mitochondria, which culminates in the organelle division [89]. DRP1 activity can be regulated by phosphorylation, ubiquitination, sumoylation, and S-nitrosylation. In particular, the phosphorylation regulates DRP1 recruitment to the mitochondria and its activation. DRP1 phosphorylation at serine 616 residue (S616) by ERK promotes its recruitment on mitochondria, while the cAMP/PKA-dependent phosphorylation of serine 637 (S637) recruits DRP1 in the cytosol, inhibiting the fission process [90]. The dephosphorylation of S637 by the Ca^2+^-dependent phosphatase calcineurin drives DRP1 mitochondrial association and fission [91].

The recruitment of DRP1 is also mediated by the mitochondrial receptor FIS1, mitochondrial dynamics proteins of 49 kDa and 51 kDa (MID49 and MID51), and the mitochondrial fission factor (MFF) [92]. The mitochondrial shape is also associated with cristae remodelling that plays a central role in the respiratory chain functionality and in the regulation of apoptosis [26,93,94]. In general mitochondrial fusion is associated with a higher energetic efficiency and increase of ATP production, whereas fission is associated with increased mitochondrial ROS production [26].

During apoptosis, mitochondria can undergo permeabilization as a result of the mitochondrial permeability transition (MPT) or the mitochondrial outer membrane permeabilization (MOMP) [95] associated with release of cytochrome c and other apoptogenic proteins to the cytosol. However, mitochondrial dynamics have been also associated with apoptosis [96], and this finding has been supported by observation of direct interaction of MFN2 with the apoptotic proteins BAK, BCL-2, and BCL-xL [97]. Apoptotic programming is associated with extensive fragmentation of mitochondria, which is not merely a consequence of cell death but it is regulated by balance of fusion/fission proteins [98]. In addition, it has been shown that DRP1 colocalizes with MFN2 and BAX in the mitochondrial outer membrane, its recruitment causes membrane remodelling, and it is associated with BAX oligomerization and cytochrome c release [99,100]. Mitochondrial morphology is also regulated by BAK. It has been shown that BAK knockout in different mouse cells results in attenuation of mitochondrial fragmentation [98]. The proteins that participate in mitochondrial fusion and fission process can define a phenotype that protects the cells from apoptosis, as shown in the condition of FIS1 and DRP1 downregulation, MFN1 and MFN2 overexpression [101] and increased levels of OPA1 [26]. In particular, enhanced OPA1 protein level can favour its own oligomerization at the cristae junction to reduce the cristae width at the junction, decreasing or delaying the release of apoptogenic molecules into the cytoplasm [26,102].

### 4.2. Alteration of Mitochondrial Dynamics in Ovarian Cancer

Considering that the balance between mitochondrial fission and fusion, as well as biogenesis and cristae morphology, promptly respond to changes in cellular metabolic requirements and ATP demand/supply, it may be expected that the changes in energetic demand of ovarian cancer cells is associated with modifications of mitochondrial dynamics and morphology. In fact, alterations in mitochondrial dynamics and structure have been reported in ovarian cancer [21,28,67]. In human ovarian cancer tissues, electron microscope analysis revealed an increase in mitochondrial number and length and a decrease in cristae width and cristae junction diameter [21] that could represent an adaptive response of mitochondria for energy supply of cancer cells [79,103]. In this context, different proteins involved in mitochondrial dynamics and structure have been found altered in OC.

OPA1, in addition to its fundamental role in mitochondrial fusion, regulates mitochondrial crista structure [104] through the oligomeric self-interaction [105]. In fact, increased levels of OPA1 protein associated with the increase in mitochondrial length, and thus fusion process, have been reported in ovarian cancer tissue [21]. In addition, an increased level of OPA1, favouring its own oligomerization at the cristae junction, is in agreement with the reduction of the cristae width at the junction reported in OC [21]. The decrease of cristae width can be also associated with an increase of mitochondrial respiratory chain activity that could result in greater energy demand for cellular growth. On the other hands, this characteristic morphologic aspect of cristae junction reduction supports a decreased and delayed release of apoptogenic molecules into the cytoplasm following cell death stimulus [106,107] thus representing a factor indicating resistance to apoptosis in ovarian cancer tissue. The antiapoptotic role of OPA is also due to its proteolytic processing. The protease activity of OMA1, a protease that cleaves OPA1, is inhibited by PHB2/stomatin-like protein 2 complex (STOML2). In mitochondria, PHB2 and STOML2 are anchored to the mitochondrial inner membrane to regulate mitochondrial protease activity [108]. PHB2 and STOML2 are overexpressed in OC [21,109] and the destruction of the STOML2/PHB2 complex results in OMA1 activation [110]. Indeed, elevated levels of these proteins result in inhibition of OMA1 activity, OPA1 processing and resistance to proapoptotic stimuli.

Recently, a high level of OPA3 protein has been reported in ovarian cancer tissues and cells [111]. The OPA3 gene was first identified in patients with optic neuropathy, and encodes for an OPA3 protein that is a mitochondrial protein involved in the shape and structure of the mitochondria [112]. The high expression of OPA3 mRNA and protein levels in OC are associated with poor prognosis [111]. Importantly, it was reported that OPA3 inactivation increased sensitivity of ovarian cancer cells to PFI-1 and WZ4003 antiproliferative drugs [111].

MFN1 and MFN2 are responsible for mitochondrial outer membrane fusion [113]. MFN1 interacts physically with MFN2 and with OPA1 [114]. Furthermore, antiapoptotic protein BCL2 has been shown to interact with MFN2 in promoting mitochondrial fusion, and cell survival in OC [115]. MFN2 stability is also regulated by cystathionine b-synthase (CBS) an enzyme that catalyses the condensation of L-serine with homocysteine to generate the thiol ester cystathionine, an intermediate step in the production of cysteine. Clinically, OC patients harbouring increased expression of CBS and MFN2 have a poor prognosis [115]. Furthermore, in ovarian cancer cells the inhibition of CBS results in oxidative stress conditions, activating JNK that in turn phosphorylates MFN2 and results in its degradation [115]. This is associated with fragmentation of mitochondria, decreased respiration and ATP production. The supplementation of OC cells with hydrogen sulfide or gluthatione (the products of CBS activity) restored the expression of MFN2 improving mitochondrial morphology and sustaining tumours cell proliferation [115]. On the other hand, another study reported that OC patients with higher MFN2 expression had better survival than those with lower MFN2 levels and pharmacological or genetic activation of MFN2 leading to mitochondrial fusion and decreased ROS generation, resulting in reduced cell proliferation [116].

Different studies in different models of OC have pointed out a pro-fusion equilibrium leading to an increase of mitochondrial length associated with an anti-apoptotic structure of cristae [21,22,67] that probably confer to the cells an advantage for growth and proliferation. However, other studies showed a pro-fission equilibrium. Indeed, in an OVCA420 cell model, increased expression of the mitochondrial fission protein DRP1, associated with a loss of mitochondrial membrane potential and dependence on glycolysis, has been found [23], and DRP1 expression changed among different histological subtypes [117]. The phosphorylation of DRP1 is also altered in OC. In this regard, an increase of SIRT6 protein level has been reported in ovarian cancer tissues, priming the ERK1/2-dependent DRP1 phosphorylation at serine-616. This results in fragmented mitochondria that promote cellular invasion [118]. Nevertheless, these cells appear to be more sensitive to chemotherapy, probably due to the higher expression of DRP1 [117].

### 4.3. Mitochondrial Morphology and Chemoresistance in Ovarian Cancer

**Figure 3:**
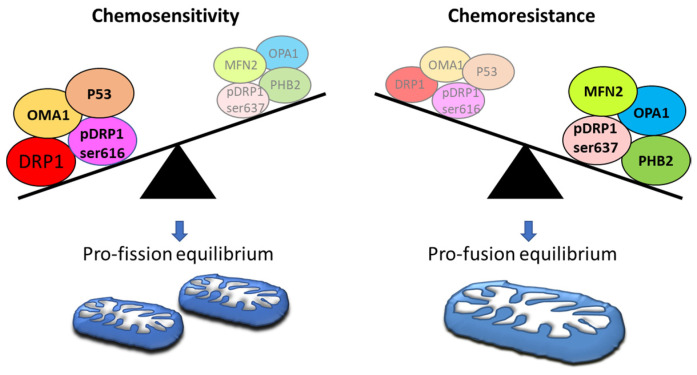
Mitochondrial dynamics protein levels in chemosensitivity and chemoresistance of ovarian cancer. Mitochondrial dynamics proteins are involved in chemosensitivity and/or chemoresistance in OC [28]. A pro-fusion equilibrium has been observed in drug-resistant cells associated with increased levels of several mitochondrial shaping proteins such as MFN2, OPA1 PHB2, and phosphorylated DRP1 at serine 637 [119,120,121,122]. On the contrary, a pro-fission equilibrium has been observed in drug-induced cell death associated with increased levels of several mitochondrial shaping proteins such as P53, activated OMA1, DRP1 and phosphorylated DRP1 at serine 616 [123,124,125,126].

Mitochondrial dynamics, structure and, in particular, mitochondrial shaping proteins appear to be responsible for the chemosensitivity and/or chemoresistance (Figure 3). Accordingly, decreased mitochondrial fission and/or increased fusion have been shown to be associated with chemoresistance in all gynaecological cancers, including ovarian cancers [28].

OPA1 is also involved in chemoresistance. Studies in ovarian cancer cell cultures showed that the chemoresistance to some drugs such as cisplatin, the first platinum-based complex to treat patients with OC, is partly due to a deregulation of OPA1 processing [119], which results in an increase of mitochondrial fusion and decreased apoptosis [28].

Furthermore, the activation of OMA1, the protease that cleaves OPA1, increased OC sensitivity to cisplatin in vivo and in vitro. Indeed, cisplatin-activating OMA1, induces L-OPA1 processing and mitochondrial fragmentation in chemosensitive cells, and this does not occur in chemoresistant ovarian cancer cells. The chemosensitivity to cisplatin is mediated by p53. In fact, its silencing inhibited activation of OMA1, L-OPA1 processing, mitochondrial fragmentation, and apoptosis [119]. OPA1 processing in ovarian cancer cells is also mediated by PHB1. Knock-down of PHB1 prevents cisplatin mediated activation of OMA 1 and OPA1 processing. PHB1 supports the interaction among phosphorylated p53, PHB1 and BAK, and, in turn, favours mitochondrial fragmentation [120]. Recently, the mitochondrial protease OMA1, which regulates internal and external signals in mitochondria by cleaving mitochondrial proteins, has been shown to be related to tumour progression [121]. DRP1 and MFN2 deregulation is also involved in ovarian cancer cisplatin resistance. In SKOV3 cisplatin-resistant cells, the mitochondrial fission protein DRP1 is down-regulated, while the mitochondrial fusion protein MFN2 is up-regulated. In accordance with the expression of DRP1 and MFN2, the average mitochondrial length was significantly increased in these cells, supporting again that mitochondrial dynamics contribute to the development of cisplatin resistance in ovarian cancer cells [122]. This was also shown by the silencing of DRP1 or overexpression of MFN2 that promote the resistance of SVOK3 cells to cisplatin [122]. In addition, the pro-fission activity of DRP1, as mentioned before, depends on its phosphorylation status. In particular, the dephosphorylation at ser637 is a pro-fission event. An in vitro study on OC cell cultures showed that the dephosphorylation at ser 637 of DRP1 increases mitochondrial fission conferring more sensitivity to cisplatin [122].

It has been found that saikosaponin-d, a saponin from a herbal plant extract, induced mitochondria fragmentation via decrease of phospho-Ser637-Drp1 in chemoresistant OVCA cells, sensitizes these cells to cisplatin [123]. Other studies have confirmed that cisplatin or paclitaxel induce ovarian cancer cell death by down-regulation of DRP1 phosphorylation at serine 637, enhancing mitochondrial fragmentation [124]. Related to the importance of DRP1 in response to chemotherapy in ovarian cancer, several studies have evaluated the possibility of use, in combination with chemotherapy, of various phytochemicals such as piperlongumine, piceatannol, and sambucus nigra agglutinin, that can induce mitochondrial fission by decreasing DRP1 phosphorylation Ser637 and increasing DRP1 and FIS1 mRNA levels [124,125,126].

Summarizing, the sensitivity of ovarian cancer cells to chemotherapeutic drugs involves p53 phosphorylation via OPA1 processing or by DRP1ser637 dephosphorylation [28], both leading to mitochondrial fragmentation [123,124,125,126].

### 4.4. Underscoring the Possible Importance of cAMP/PKA Signalling in Regulation of Mitochondrial Dynamics in OC

**Figure 4:**
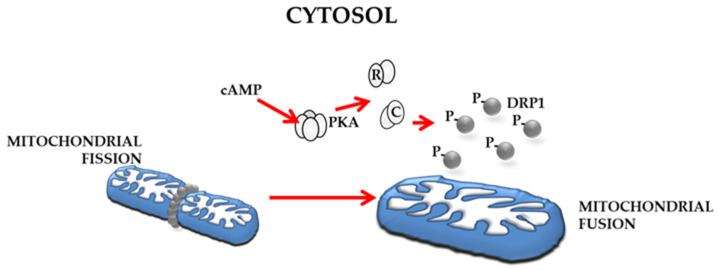
Cytosolic cAMP-dependent control of mitochondrial dynamics. Dephosphorylated form of DRP1 at serine 637 localizes in mitochondria forming a ring surrounding mitochondrial outer membrane and promoting fission event. Activation of cAMP cascade promotes the phosphorylation of DRP1 at serine 637, its delocalization in the cytoplasm and thus promoting fusion event. cAMP level is augmented in OC [21]. The expression of catalytic subunit (C) of PKA and increased mRNA of regulatory subunit (R) are correlated with advanced stage and more aggressive ovarian cancer disease [143,144,145,146,147].

Cell growth is supported by several signalling pathways, and the onset and progression of the tumour is associated with deregulation of several signal transduction pathways. Post-translational processes, including phosphorylation, ubiquitination, methylation, and acetylation, are involved in control of cell signal transduction pathways. Protein kinases and phosphatases are often abnormally or uncontrollably activated in cancers, so much so they are the prime candidates for molecularly targeted therapies [127,128,129,130]. Cellular pathways are often triggered by signal molecules, such as those produced by growth factors, hormones or ions; among others, cyclic AMP (cAMP) is the most studied [131,132]. Second messenger cAMP can modulate a large number of physiological processes, including gene expression, metabolism, channel activation, cell proliferation and differentiation, and cell death [133]. In mammalian cells, cAMP can be synthesized by the trans-membrane subfamily of adenylyl cyclase (tmAC), or by soluble adenylyl cyclase (sAC) localized within the cytosol, mitochondria and nucleus [134]. PKA and EPAC represent targeting down-stream effectors, and have received a lot of attention in cancer research [135]. The cAMP/PKA pathway modulates several mitochondrial processes such as mitochondrial respiratory chain activity and organization [136,137,138], dynamics [69] and also mitochondrial mediated apoptosis [139,140]. Deregulation of the cAMP signal has been shown in several tumours, such as colorectal cancer, glioblastoma, breast cancer and OC [135]. Hypoxic activation of cAMP/PKA pathway has been reported in different lines of cancer cells [141,142], and several studies have reported an involvement of PKA deregulation in OC. The expression of catalytic subunit of PKA and increased mRNA of regulatory subunit (RIα -PKA) were found to be correlated with advanced stage and more aggressive ovarian cancer disease (Figure 4) [143,144,145,146,147]. In addition, increased PKA activity and its subcellular localization by A-Kinase anchoring proteins (AKAPs) are involved in cell migration in SKOV-3 cells. Inhibition of either PKA activity and AKAP-mediated PKA anchoring blocks invasion suggests a role of PKA in ovarian cancer metastasis [147]. Furthermore, an increased cAMP level associated with PKA activation has been reported in ovarian cancer tissues [22].

**Figure 5:**
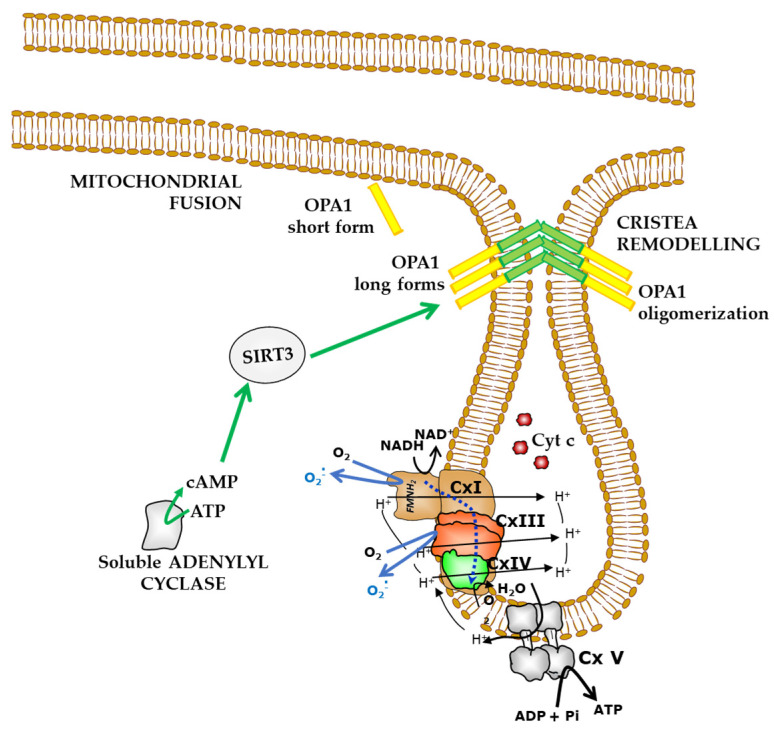
Mitochondrial cAMP-dependent control of mitochondrial dynamics. Soluble adenylyl cyclase produces cAMP inside mitochondria. Mitochondrial cAMP determines fusion event by sustaining SIRT3 protein level that, in turn, deacetylates OPA1 inhibiting its degradation from L to S-forms [21]. OPA1 oligomerization at the inner mitochondrial membrane keeping the cristae junctions tight and favors the fusion event. This prevents the release of cytochrome c (Cyt c) making the cells more resistant to apoptosis. Deregulation of OPA1 protein level and proteolytic processes has been found in OC [119].

Deregulation of cAMP/PKA in ovarian cancer tissue could be involved in mitochondrial dynamics deregulation, interfering with proteins involved in dynamic machinery. Indeed, the processing of OPA1 can also be modulated by the signal pathway mediated by the mitochondrial cAMP (mt-cAMP)/PKA (Figure 5) [69]. In fact, a decrease of mt-cAMP level activates mitochondrial proteases that, in turn, can cause a decrease of SIRT3 protein level. This results in hyperacetylation of OPA, promoting its processing and pushing the cells towards apoptosis [62]. In agreement with this, sustained cAMP level results in an increased SIRT3 protein expression [148]. Signorile et al. have argued that PKA activation in ovarian cancer tissues could affect mitochondrial dynamics and apoptosis resistance by stabilization of the SIRT3 protein and inhibition of OPA1 processing [22,62]. Furthermore, studies conducted on ovarian cancer cell cultures have shown that chemoresistance to some drugs is partly due to a deregulation of OPA1 processing [119]. Also SIRT3 can be considered a tumour promoter or suppressor based on cell type [149,150]. It has been reported that decreased levels of SIRT3 promote metastasis of OC [151], while the induction of apoptosis in SKOV3 ovarian cancer cells is related to the activation of SIRT3 [152].

The cAMP/PKA pathway can modulate mitochondrial dynamics in OC by controlling OPA1 processing (via SIRT3) (Figure 5) [21], and it could also promote the PKA-phosphorylation of DRP1 at serine 637 (Figure 4) [153].

## 5. Conclusions

Numerous studies have revealed that several aspects of mitochondria are extensively implicated in OC. Mitochondrial function can be upregulated in some ovarian cancer cells, potentially rendering these tumours more sensitive to respiratory chain complex inhibition [154]. Deregulation of mitochondrial dynamics and apoptosis represents another key point for the onset, progression and chemoresistance of cancer. In OC, due to the imbalance between mitochondrial fission and fusion, changes of mitochondrial morphology occur [155]. Mitochondrial dynamics appear to be strongly involved in OC, and also contribute to chemosensitivity and chemoresistance. The complex machinery of mitochondrial dynamics includes many proteins and signalling pathways. Thus, comprehension of their molecular mechanisms could be useful for stratification of patients identifying specific cancer types and/or molecular characteristics, and it may also be useful for selecting new ‘druggable’ targets to prevent treatment failure and improve prognosis.

## Data Availability

Not applicable.
